# Expedited diagnosis of disseminated *Mycobacterium kansasii* infection using targeted (amplicon-based) next-generation sequencing in an immunocompromised patient

**DOI:** 10.1128/asmcr.00110-24

**Published:** 2025-09-08

**Authors:** Risa Fuller, Bruce E. Petersen, Shafinaz Hussein, Christian Salib, Amy Duffield, Rebecca Gaglia, Janice L. Gabrilove, Carolina Hernández, Juan David Ramirez, Samantha E. Jacobs, Alberto Paniz-Mondolfi

**Affiliations:** 1 Department of Medicine, Division of Infectious Diseases, Icahn School of Medicine at Mount Sinai 377569, New York, New York, USA; 2 Department of Pathology, Molecular, and Cell-Based Medicine, Molecular Microbiology Laboratory, Icahn School of Medicine at Mount Sinai 5925, New York, New York, USA; 3 Tisch Cancer Institute, Icahn School of Medicine at Mount Sinai 145753, New York, New York, USA; Pattern Bioscience, Austin, Texas, USA

**Keywords:** *Mycobacterium kansasii*, disseminated, T-cell large granular lymphocytic leukemia, metagenomics, next-generation sequencing, diagnostics

## Abstract

**Background:**

*Mycobacterium kansasii* is typically associated with pulmonary disease and is an uncommon cause of disseminated infection and thus can be challenging to diagnose and treat.

**Case Summary:**

We present a 59-year-old female with a history of renal cell carcinoma (RCC) and T-cell large granular lymphocytic leukemia (T-LGLL) who developed disseminated *Mycobacterium kansasii* infection. Targeted next-generation sequencing (tNGS) facilitated the rapid detection of *M. kansasii* from formalin-fixed, paraffin-embedded (FFPE) tissue, aiding in clinical decision-making prior to culture confirmation.

**Conclusion:**

The case highlights the diagnostic challenges posed by overlapping clinical features and chronic granulomatous inflammation in immunocompromised patients, as well as the utility of amplicon-based sequencing in expediting diagnostic turnaround and guiding therapeutic interventions.

## INTRODUCTION

Non-tuberculous mycobacteria (NTM) are increasingly recognized pathogens in immunocompromised patients. Among these, *Mycobacterium kansasii* is typically associated with pulmonary disease and is an uncommon cause of disseminated infection (1). Diagnosis can be especially challenging due to overlapping signs and symptoms with other infectious and non-infectious conditions and the frequent need for invasive procedures to obtain microbiologic confirmation (2). This often leads to delays in appropriate therapy and worse patient outcomes (2).

This report presents a case in which disseminated *M. kansasii* infection was diagnosed through targeted next-generation sequencing (NGS) on formalin-fixed, paraffin-embedded (FFPE) tissue, an advanced technique that rapidly identified the pathogen in a complex clinical scenario. NGS allowed for pathogen identification well before confirmatory results from traditional tissue culture and matrix-assisted laser desorption/ionization time-of-flight (MALDI-ToF: MALDI Library: Bruker MBT Mycobacteria Library we used is version 7) were available, demonstrating the clinical utility of sequencing technologies in guiding timely diagnosis and treatment.

## CASE PRESENTATION

A 59-year-old woman initially presented 3 years ago with hepatosplenomegaly and a renal mass, later diagnosed as renal cell carcinoma (RCC) and surgically resected. A bone marrow biopsy revealed a hypercellular marrow with non-necrotizing granulomatous inflammation (negative for acid-fast bacilli and fungi by AFB: in-house Ziehl-Neelsen for FFPE and Grocott’s Methenamine Silver (GMS) tissue stains, and a clonal expansion of T-cell receptor (TCR) gamma gene rearrangement+, CD3+CD8+/CD57+/CD56− T-cell population consistent with LGLL. Peripheral blood flow cytometric analysis and TCR gamma gene rearrangement studies confirmed this diagnosis.

Over the subsequent year, she had recurrent episodes of fatigue, mental cloudiness, pancytopenia, and hypercalcemia. A liver biopsy 10 months later showed involvement by T-LGLL in addition to non-necrotizing granulomas (negative for acid-fast organisms and fungi by Ziehl-Neelsen, Fite-Faraco, GMS tissue stains), suggestive of hepatic sarcoidosis. One month later, she was started on cyclophosphamide and prednisone for T-LGLL, but this was discontinued after 5 months due to the development of neutropenic fever. Weekly methotrexate was initiated 1 month later, resulting in partial symptomatic improvement.

A positron emission technology (PET) scan 2 years after initial presentation revealed stable hepatosplenomegaly, a new hypermetabolic lymph node mass suggestive of malignancy, and increased metabolic activity in select pelvic bone regions. She continued reporting intermittent fatigue, fever, and chills, and another PET 9 months later showed stable hepatosplenomegaly and decreased lymphadenopathy. A subsequent bone marrow biopsy revealed persistence of an atypical T-cell population but no granulomatous disease.

Two months later, after returning from a Caribbean cruise, she developed right foot pain and was diagnosed with a stress fracture. The following month, she developed swelling and pain of the left third toe that did not improve with empiric doxycycline or ciprofloxacin. Magnetic resonance imaging (MRI) of the left foot indicated an “aggressive” soft tissue mass causing osseous destruction of the middle phalanx and distal phalanx base of left third toe. A PET scan showed stable lymphadenopathy with multiple new fluorodeoxyglucose (FDG)-avid lesions through the musculature and soft tissue in the lower extremities and right elbow. A biopsy of the toe showed acute and chronic inflammation associated with granulation tissue; the sample was not sent for culture.

**Fig 1:**
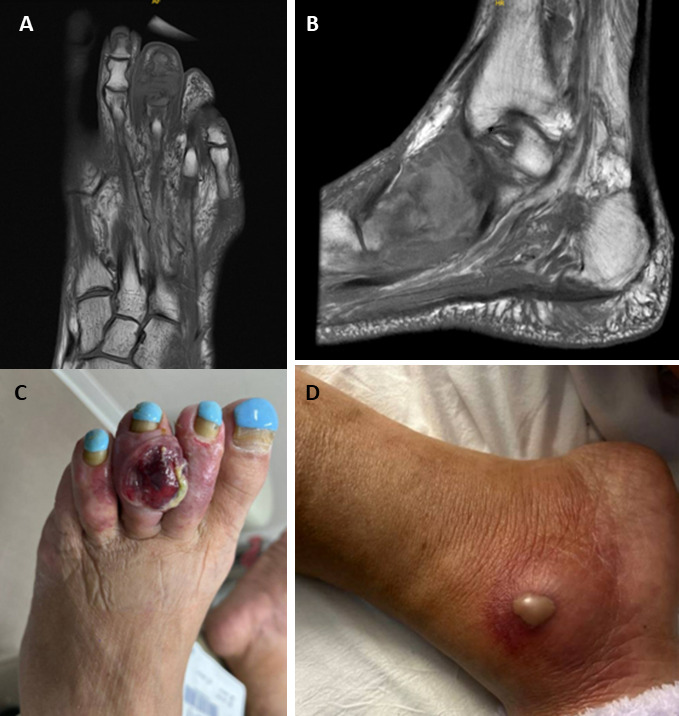
(**A**) MRI of left foot showing third toe soft tissue mass and osseous destruction of middle phalanx and distal phalanx base. (**B**) Left third toe lesion. (**C**) MRI of right foot showing large heterogeneous mass with extensive destruction of adjacent bones. (**D**) Right ankle lesion.

She developed high fevers (103°F) and worsening pain in the right ankle, prompting admission to the hospital. On physical examination, there was a painful ulcerative mass on the dorsal aspect of the left third toe, an area of erythema and fluctuance with purulent drainage on the right medial malleolus, and several small non-tender subcutaneous nodules on the legs and right elbow (Fig. 1). Labs were as follows: WBC 3.8 × 10^3^ /μL (ref 3.4–10.8 × 10^3^/μL), hemoglobin 7.7 g/dL (ref 11.1–15.9 g/dL), platelets 114 × 10^3^/μL (ref 150–379 × 10^3^/μL), and total bilirubin 1.8 mg/dL (ref 0.0–1.2 mg/dL). She was empirically started on intravenous vancomycin 1.25 g every 12 h and cefepime 1 g every 8 h. MRI of her right foot revealed a large heterogeneous enhancing mass in the medial aspect of the right ankle with destruction of the adjacent navicular and first and second cuneiform bones (Fig. 1). She continued to have daily fevers up to 105°F.

**Fig 2:**
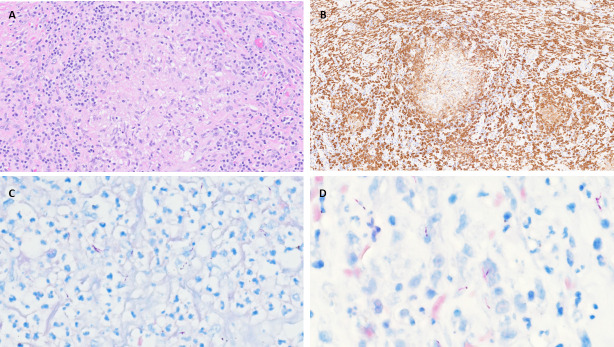
Histological sections showcasing granulomatous inflammation stained with hematoxylin & eosin (**A**), immunostaining highlighting CD163-positive macrophage infiltration (**B**), and mycobacteria with a classic elongated, beaded morphology on FITE (**C**) and acid-fast (**D**) stains.

A bone marrow biopsy was notable for a hypercellular marrow with non-necrotizing granulomatous inflammation and GMS (Grocott’s Methenamine Silver) and AFB (in-house Ziehl-Neelsen for FFPE) stains of tissue were negative for microorganisms. An incisional biopsy of the left buttock mass revealed extensive acute and chronic inflammation with non-necrotizing granulomas. GMS, AFB, and FITE (Fite-Faraco) tissue stains were positive for rod-shaped microorganisms and negative for carcinoma (Fig. 2). A retrospective Ziehl-Neelsen stain on the prior left biopsy was also positive for acid-fast organisms reported indeterminate for GMS, Gram, and Steiner stains. She was evaluated by rheumatology and pulmonology for possible sarcoidosis, but this was felt to be unlikely given the clinical presentation and positive AFB staining of multiple tissue samples. Serologic testing for fungal pathogens, including *Coccidioides* IgM and IgG antibody, *Blastomyces* antibody, and cryptococcal antigen, and urine histoplasma antigen, was all negative. Due to the high pre-test suspicion for malignancy, these samples were not sent for culture. Given the extensive granulomatous inflammation, systemic signs of infection, positive AFB staining, and lack of response to routine antibiotics, disseminated mycobacterial infection was suspected. Consequently, offline molecular testing was pursued on FFPE tissue samples using a research in-house targeted next-generation sequencing (NGS) assay. This assay is for research use only (RUO) and has not been validated for clinical diagnostic use.

For research *in-house* targeted NGS assay, DNA extraction was conducted using the QIAamp DNA FFPE Tissue Kit (Qiagen, 56404). The 16S-rRNA gene was amplified for genus-level bacterial and archaeal identification using primers 27F (AGAGTTTGATCMTGGCTCAG) and 1492R (GGTTACCTTGTTACGACTT). A sequencing library was prepared with the Ligation Sequencing Amplicons – Native Barcoding Kit (SQK-NBD114.96, Oxford Nanopore Technologies). PCR products underwent end-repair, A-tailing, barcode ligation, and adapter ligation using NEB kits. The library was then sequenced on a MinION Mk1C device with an R10.4.1 flow cell (FLO-MIN114). Base calling was completed using Dorado v0.7.2 with a filter for reads below a quality score of 8, while quality assessment through Nanostat and SeqKit confirmed a minimum score of 10 for filtering. Taxonomic assignment was performed using EMU with the Silva Database. ZymoBIOMICS Microbial Community Standard was used as a positive control to verify the detection of the described microbial communities, and water, tested for genomic DNA contamination, as a negative control, ensuring no more than 100 assigned reads were obtained.

Taxonomic analysis revealed that the most abundant genera were *Bacteroides*, *Pseudomonas*, and *Streptococcus. Bacteroides fragilis* was the most prevalent at 40.2%, with an estimated 33,371 reads, followed by *Pseudomonas aeruginosa* at 7.3% (6,109 reads) and *Streptococcus salivarius* at 2.8% (2,322 reads), suggesting a mix of gastrointestinal and skin microbiota. Although *Mycobacterium kansasii* was identified at a low abundance of 0.25% with an estimated 206 reads, this finding aligned with the histopathological results. Additionally, the patient did not have any improvement on broad-spectrum antibiotics for more than 1 week, suggesting that non-AFB bacteria were less likely the causative pathogens.

Given limited tissue availability for susceptibility testing, a subsequent biopsy of the left third toe was performed 2 weeks after admission. This biopsy revealed numerous acid-fast bacilli, later confirmed as *Mycobacterium kansasii* through culture and MALDI-ToF identification after 11 days of incubation. The maximum score was 2.14 on Bruker MALDI Biotyper RUO System, using the Bruker MBT *Mycobacteria* Library, and prepared using bead extraction method from MGIT broth. The tissue also grew *Pseudomonas aeruginosa, Proteus mirabilis, and Bacteroides fragilis*, thought to be consistent with colonization of an open foot wound given the lack of improvement on broad-spectrum antibiotics and histopathology results suggesting mycobacterial disease. The patient was discharged after clinical stabilization. She was initiated on rifampin, azithromycin, and ethambutol on follow-up 2 weeks later. The systemic symptoms of fever, malaise, anorexia, and fatigue, as well as the left toe and right lateral malleolus ulcerations, improved, though the cutaneous lesions continued to wax and wane. Therefore, approximately 5 months later, IV amikacin 15 mg/kg three times weekly was added to her regimen, and she experienced significant improvement. The patient was referred to immunology and is undergoing evaluation for primary and acquired immunodeficiency. Genetic evaluation for chronic granulomatous disease was negative.

## DISCUSSION

*Mycobacterium kansasii* is a slow-growing acid-fast mycobacterium that can take up to 6 weeks to grow in culture and produces characteristic yellow colonies when exposed to light (Runyon I) (1). *M. kansasii* is the sixth most commonly isolated NTM species globally, with a prevalence of 9.4% among NTM species and the highest prevalence of *M*. *kansasii* reported in Europe (3).

*M. kansasii* typically presents as a pulmonary infection and is a rare cause of disseminated infection (2, 3). A patient with skin lesions due to *M. kansasii* was first reported in the literature in 1995 (4). Since, there have been several published cases of *M. kansasii* cutaneous infection (2, 5–7), though most patients present with pulmonary disease, lymphadenopathy, or intra-abdominal abscesses (5, 6, 8–14). Most reported cases are in patients with some significant form of immunodeficiency, with a strong association between disseminated infection and anti-interferon-gamma antibodies (7, 11, 14).

Our patient had RCC and CD3+, CD8+, CD57+, CD56− T-LGLL and was not known to be severely immunocompromised; hence, she requires investigation for conditions that may predispose her to disseminated NTM infection. Additionally, her prolonged granulomatous inflammation, initially attributed to LGL or immune dysregulation, might have been related to a low-grade *M. kansasii* infection, although such persistence without overt symptoms for years would be unusual.

While other microorganisms were detected in the tissue sample, the clinical presentation was not consistent with a typical bacterial infection. The chronicity, pathologic findings, waxing and waning nature of the infection, and multitude of sites of infection were most consistent with mycobacterial infection.

From a diagnostic perspective, this case underscores the utility of tNGS in identifying complex infections. Here, an NGS approach enabled the retrospective detection of the pathogen in a challenging FFPE tissue matrix, where low pathogen biomass, nucleic acid fragmentation, and other sequencing artifacts often complicate analysis. This approach provided actionable clinical information prior to identification via traditional methods (culture and MALDI-ToF). Despite the inherent challenges of a metagenomic approach—often likened to finding a “needle in a haystack” — this case highlights the utility of NGS for improved pathogen detection when interpreted within the appropriate clinical and histologic contexts. We do recognize the limitations, however. In this case, culture data corroborated our findings, but that is not always the case, and this can make diagnosis challenging, especially when more than one organism is identified. Furthermore, cultures are still often needed for susceptibility testing to guide treatment.

Next-generation sequencing (NGS) methods, such as Nanopore, as used in this case, demonstrate strong potential to enhance diagnostic performance by enabling the accurate identification of diverse microbial populations and reducing turnaround times, ultimately contributing to improved clinical care. Nonetheless, rigorous validation is essential before these technologies can be reliably implemented in clinical practice. As bioinformatic algorithms for pathogen detection in clinical contexts continue to advance, the successful integration of NGS-based diagnostics relies on a multidisciplinary approach that brings together clinicians, microbiologists, and pathologists to support informed decision-making and optimize patient outcomes.

### Conclusion

This case illustrates the valuable role of metagenomic sequencing as a complementary tool in complex diagnostic scenarios, especially for patients with challenging clinical presentations and limited tissue samples. NGS enabled the early detection of *Mycobacterium kansasii* from FFPE tissue, providing critical insights before confirmatory culture and MALDI-ToF results were available. By integrating metagenomic data with clinical, histopathological, and microbiological findings, the diagnostic process was accelerated, allowing for a timely and targeted therapeutic approach. The success of this case highlights how a multidisciplinary framework—involving clinicians, pathologists, and microbiologists—can enhance diagnostic accuracy and patient outcomes, particularly when conventional methods alone may be insufficient.
